# Supplementary material for: SBML qualitative models: a model representation format and infrastructure to foster interactions between qualitative modelling formalisms and tools
Source: BMC Syst Biol. 2013 Dec 10;7:135. doi: 10.1186/1752-0509-7-135 (PMC3892043; doi:10.1186/1752-0509-7-135)
Supplement: Additional file 1 — Complete dynamic profile of the example model. The model was simulated with consistent results in the Cell Collective (left column of panels A & B), GINsim (right column of panels A & B), and CellNOpt (data not shown, but simulations were consistent with those presented here). Charts at the top of the Cell Collective column correspond to the overall dynamic profile across all nodes in the model. Black cells correspond to active (1) states, whereas inactive (0) states are white. The bottom graphs in the Cell Collective column illustrate the time course of selected nodes. The GINsim columns show State Transition Graph and the Hierarchical Transition Graph (HTG) generated with the tool. Note that due to the synchronous updating, the irreversible components of the (HTG) correspond to linear chains of states. Simulations were performed under four input conditions: EGF = TNFα = 0; EGF = TNFα = 1; EGF = 0 & TNFα = 1; EGF = 1 & TNFα = 0. A) EGF = TNFa = 0. The network reaches a steady state (shown in both GINsim and the Cell Collective column) after 3 transient states. The order of the individual species states in the steady state generated by GINsim is sorted in the same (alphabetical) order, as presented in the Cell Collective column. B) EGF = 1, TNFa = 0. The network reaches a steady state after 14 transient states. C) EGF = 0, TNFa = 1. Following 5 transient states, the network reaches a 6-cycle attractor. D) EGF = TNFa = 1. After 12 transient states, the network reaches a cyclical attractor encompassing six states. [file 1752-0509-7-135-S1.pdf]

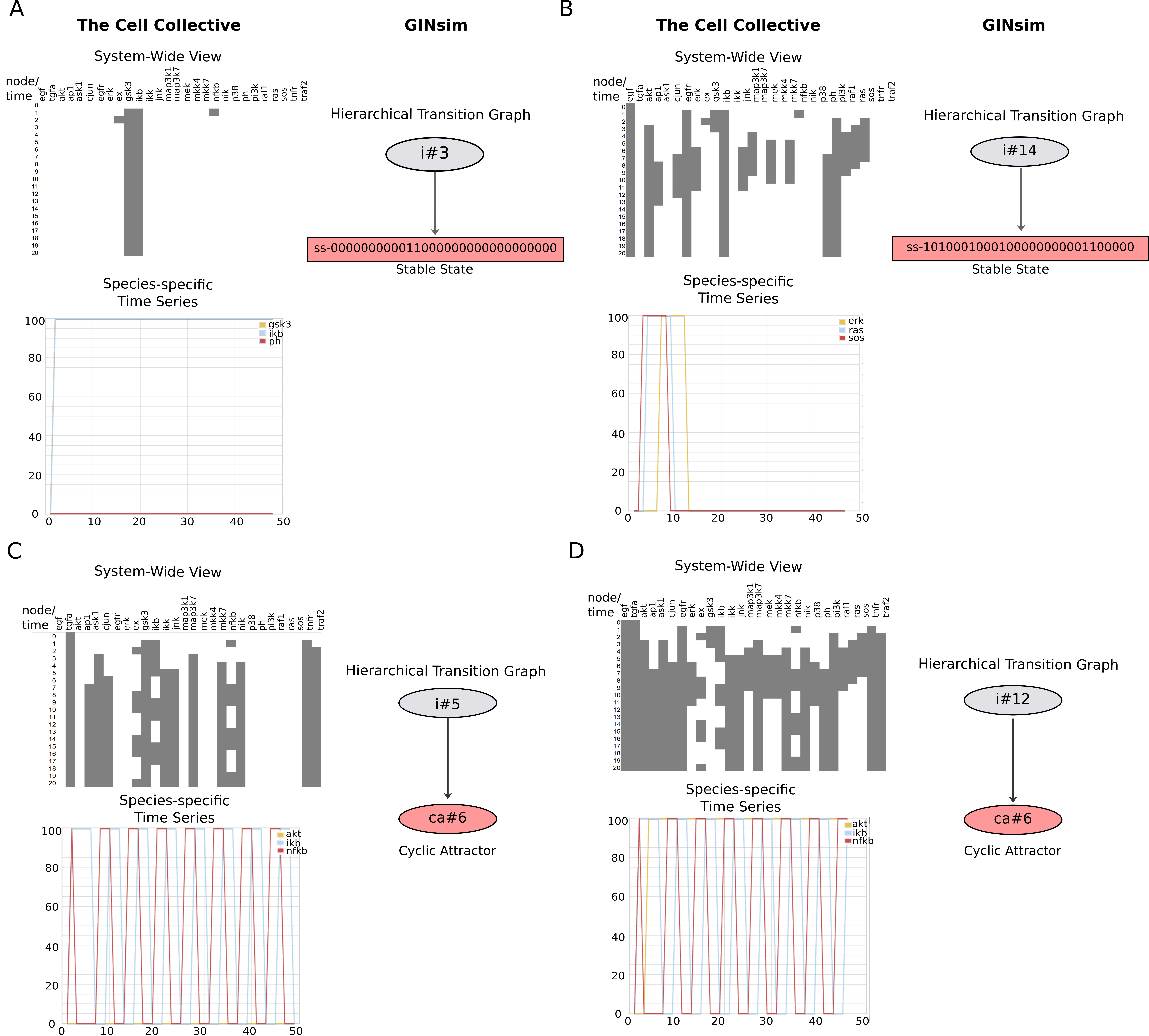

A
The Cell Collective
GINsim
System-Wide View
node/ time
egf tgfa akt ap1 ask1 cjun egfr erk ex gsk3 ikb ikk jnk map3k1 map3k7 mek mkk4 mkk7 nfkb nik p38 ph pi3k raf1 ras sos tnfr traf2
Hierarchical Transition Graph
i#3
ss-0000000001100000000000000000
Stable State
Species-specific Time Series
gsk3
ikb
ph
100 80 60 40 20 0
0 10 20 30 40 50
B
The Cell Collective
GINsim
System-Wide View
node/ time
egf tgfa akt ap1 ask1 cjun egfr erk ex gsk3 ikb ikk jnk map3k1 map3k7 mek mkk4 mkk7 nfkb nik p38 ph pi3k raf1 ras sos tnfr traf2
Hierarchical Transition Graph
i#14
ss-1010001000100000000011000000
Stable State
Species-specific Time Series
erk
ras
sos
100 80 60 40 20 0
0 10 20 30 40 50
C
System-Wide View
node/ time
egf tgfa akt ap1 ask1 cjun egfr erk ex gsk3 ikb ikk jnk map3k1 map3k7 mek mkk4 mkk7 nfkb nik p38 ph pi3k raf1 ras sos tnfr traf2
Hierarchical Transition Graph
i#5
ca#6
Cyclic Attractor
Species-specific Time Series
akt
ikb
nfkb
100 80 60 40 20 0
0 10 20 30 40 50
D
System-Wide View
node/ time
egf tgfa akt ap1 ask1 cjun egfr erk ex gsk3 ikb ikk jnk map3k1 map3k7 mek mkk4 mkk7 nfkb nik p38 ph pi3k raf1 ras sos tnfr traf2
Hierarchical Transition Graph
i#12
ca#6
Cyclic Attractor
Species-specific Time Series
akt
ikb
nfkb
100 80 60 40 20 0
0 10 20 30 40 50
